# An Unusual Presentation of a Rare Combination of Double Moiety and Vaginal Ureter Insertion

**DOI:** 10.7759/cureus.49574

**Published:** 2023-11-28

**Authors:** Yaman M. Alahmad, Ayman Elmajdoub, Ali Barah

**Affiliations:** 1 Radiology, Hamad General Hospital, Doha, QAT; 2 Interventional Radiology, Hamad General Hospital, Doha, QAT

**Keywords:** interventional radiology, percutaneous nephrostomy, ureter ectopic insertion, duplex renal collecting system, double moiety, renal colic

## Abstract

The association between a duplex renal collecting system and ectopic ureter insertion is well-established. Although congenital genitourinary abnormalities are usually detected during childhood, incidentally, or due to underlying symptoms or medical complications, a few cases might not be diagnosed/treated until adulthood. Herein, we present a case of a 43-year-old lady who came to the emergency department with acute left flank pain associated with a burning sensation during micturition for four days. Imaging showed a duplex collecting system, and findings suggestive of obstructive uropathy. The patient underwent an initial trial of left ureteroscopy that was unsuccessful. Therefore, the interventional radiology (IR) department was consulted to perform left percutaneous nephrostomy insertion with antegrade ureteric stenting, where the patient was noted to have ectopic ureter insertion into the anterior wall of the vagina by antegrade ureterogram. The patient underwent ureter re-implantation to save the kidney from further insult.

## Introduction

The association between the duplex renal collecting system and ectopic ureter insertion is well-established [1,2]. Although congenital genitourinary abnormalities are usually detected during childhood, incidentally, or due to underlying symptoms or medical complications, a few cases might not be diagnosed/treated until adulthood [3]. These adult patients often remain asymptomatic but can also present with hydroureteronephrosis, recurrent urinary tract infections (UTIs), or urinary incontinence [4,5]. Visualization of the course of duplicated ectopic ureters and the insertion of ureters is of extreme importance for treatment and surgical planning [6]. We present a case of a 43-year-old female who came to the emergency department with left flank pain and was found to have an obstructive left lower ureteric stone with a duplex renal system, thus she underwent an initial trial of left ureteroscopy was unsuccessful. Therefore, the interventional radiology (IR) department was consulted to perform left percutaneous nephrostomy insertion with antegrade ureteric stenting.

## Case presentation

**Figure 1:**
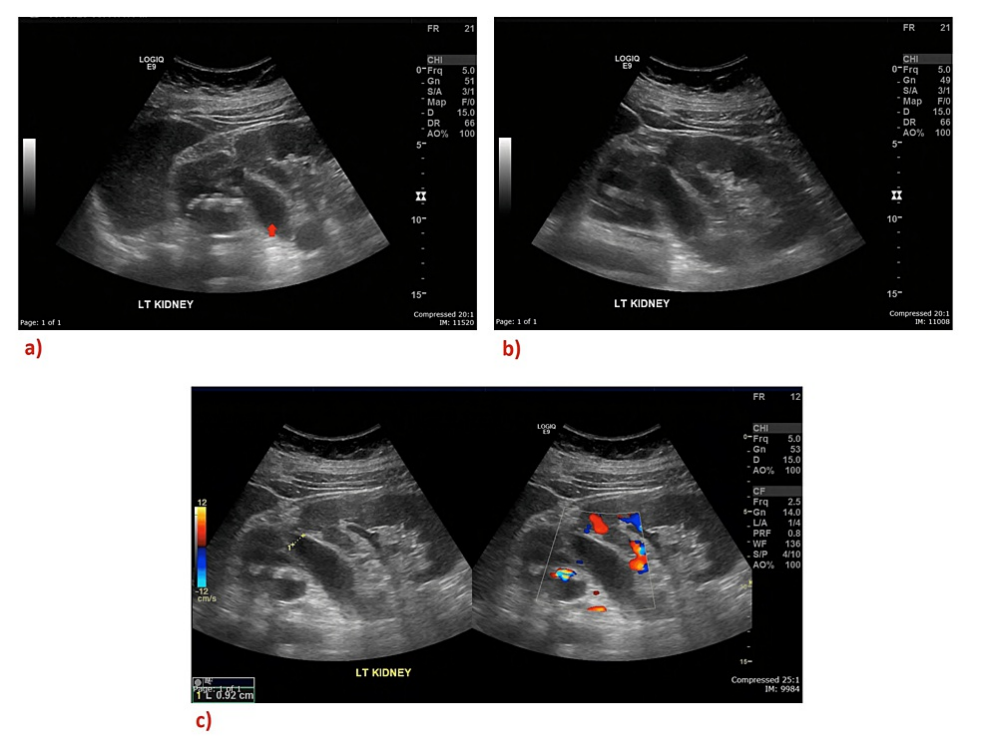
Selected images from the ultrasound abdomen and pelvis demonstrating a duplex renal collecting system with hydronephrosis and hydroureter on the left kidney Images A and B show a dilated left upper renal pelvicalyceal system (red arrow). Image C reveals the absence of color flow with a diameter of 0.92 cm, confirming a dilated pelvicalyceal system.

A 43-year-old lady presented to the emergency department with acute left flank pain associated with a burning sensation during micturition for four days. She had no fever, dysuria, hematuria, or urethral discharge. Her past medical history was only significant for occasional urinary incontinence since childhood, for which she did not seek medical advice. The initial laboratory workup was unremarkable, with normal white blood cell counts, except for elevated C-reactive protein (CRP). Urinalysis was significant for the presence of leukocytes and erythrocytes with no casts or stones. Urine culture was negative. Ultrasound abdomen and pelvis showed a duplex renal collecting system with multiple lower ureteric stones, moderate to severe upper moiety hydroureteronephrosis, and multiple stones in the upper pole of the left kidney (Figure 1).

**Figure 2:**
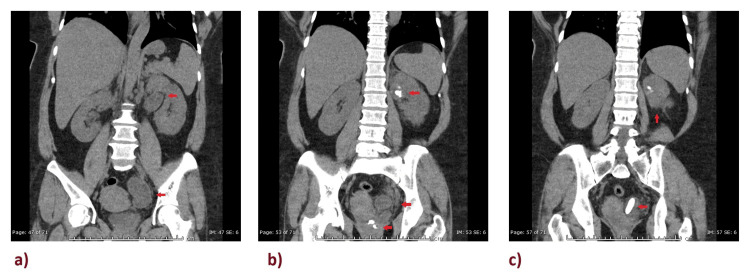
Selected coronal images from the CT urinary tract showing tortuous dilatation of the left ureter Image A reveals hydroureteronephrosis confined to the upper pole with a dilated pelvicalyceal system (red arrows). Image B shows multiple obstructive renal and ureteric calculi with significant periureteric fat stranding (red arrows). Image C points out a large obstructive distal ureteric calculus with associated left perirenal fat stranding (red arrow).

Non-contrast computed tomography (CT) of the urinary tract showed a left duplex renal collecting system with distal upper moiety ureteric stones with severe back pressure. The measurements of the largest left ureteric stone were 33 x 10 mm with attenuation value + 1133 HU, causing proximal upstream tortuous severe dilatation of the left ureter, with associated left perirenal and left periureteric fat stranding (Figure 2). The left upper renal moiety calyx shows a calculus measuring 24 x 14 mm with a CT attenuation value of + 1028 HU. A similar calculus is seen in the left renal upper moiety posterior calyx measuring 1-2 mm. 

Later, a CT urogram was performed that re-demonstrated the duplication of the left renal collecting system with ectopic insertion of the upper moiety ureter, without specifying the exact ectopic insertion site. Besides the re-demonstration of the calculus in the upper moiety, and a long calculus in the distal third of its ureter (images not shown). 

The urology team planned to perform a cystoscopy with a left ureteroscopy. However, the trial of left ureteroscopy failed as the left ureteric orifice could not be identified. Thus, an interventional radiologist was consulted to perform left percutaneous nephrostomy insertion with antegrade ureteric stenting to relieve obstructive uropathy.

**Figure 3:**
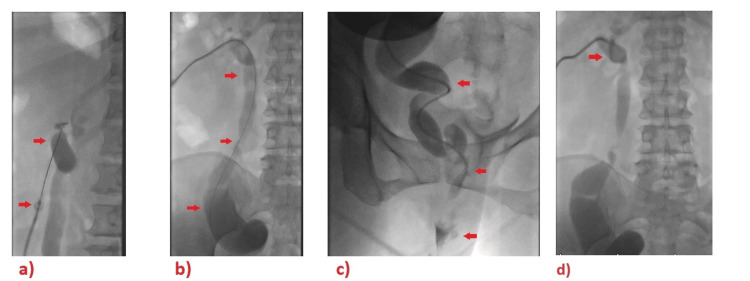
Selected images from the left ureterogram showed ectopic insertion of the left upper moiety ureter in the vagina Images A and B show the left proximal to lower tortious ureter on the ureterogram (red arrows). Image C detects an ectopic insertion of the left upper ureter/moiety into the vagina (the lowest red arrow shows contrast within the vagina). Image D shows the nephrostomy tube in place.

In the Angiosuite, the patient underwent pre-procedure ultrasound scanning, which showed a scared left kidney with minimal dilated left upper moiety. Under an aseptic technique and local anesthesia, while the patient was in the prone position, using a 21-gauge needle (AccuStick Introducer System, Boston Scientific, Marlborough, Massachusetts, USA), the left kidney (left upper moiety) was punctured from the posterior middle calyx under ultrasound guidance and then the introducer kit of the AccuStick needle was placed. After that, a Terumo guide wire 0.035 in (Terumo Corporation, Shibuya City, Tokyo, Japan) was advanced into the renal pelvis, and it went down the ureter under fluoroscopy. After tract dilation, the nephrostomy tube was inserted over the guide wire. The left ureterogram showed ectopic insertion of the left upper moiety ureter in the vagina (Figure 3). Then, an 8.5 F x 25 cm Resolve drainage catheter (Merit Medical Systems, Inc., South Jordan, Utah, USA) was inserted into the left upper moiety.

**Figure 4:**
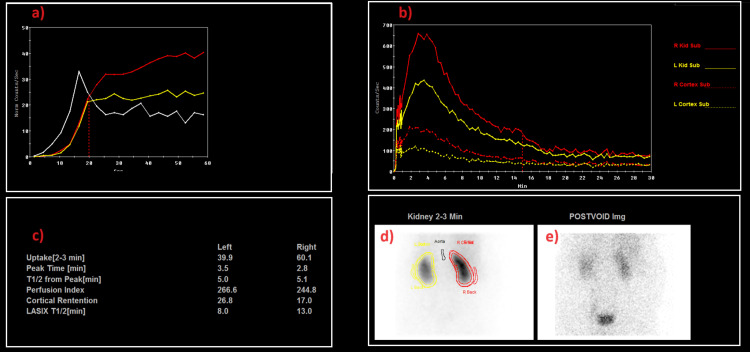
Demonstrates the outcomes of the MAG3 renogram study Image A shows patent renal perfusion bilaterally. Image B shows the non-obstructive normal renal function pattern. Image C shows the split renal function of each side, with reduced left kidney function (39.9%). Image D shows the kidneys in the normal position. The left kidney has slightly reduced cortical radiotracer uptake. Image E shows normal emptying of the radiotracer from the upper urinary tract and ureters and partial filling of the urinary bladder.

A nuclear medicine renogram was pursued to see if the left upper moiety was functioning; it showed the left kidney contributed to 39.9% of total renal function. Further, the split function of the upper and lower moiety was: left upper moiety 49.6% and left lower Moiety 50.4% (Figure 4). In other words, the uptake was only mildly reduced on the left side. The upper and lower moieties contributed equally to the left duplex system. The drainage is normal bilaterally, and there is no evidence of outflow obstruction. The patient underwent surgery for a left lower ureteric stone in the left upper moiety (completed duplex) with ectopic insertion of the left upper moiety into the anterior vaginal wall for re-implantation. There were no intraoperative complications, and the postoperative course was unremarkable.

## Discussion

Although the duplex renal collecting system is a relatively common congenital abnormality, the co-existence of ectopic ureter insertion in the vagina is rare. In females, the commonest sites of ectopic ureter insertion are the bladder neck/urethra (35%), the vestibule (30%), the vagina (25%), and the uterus (5%) [7]. Almost 80% of ectopic ureter cases are associated with a duplex renal collecting system, while in the minority of cases, a single system is found [7]. Furthermore, such anomalies are typically diagnosed during infancy and childhood via sonography or micturition cystourethrogram rather than adulthood [8]. Patients can be asymptomatic or misdiagnosed until advanced health care is considered for medical complications such as urinary tract stone formation, UTIs, and/or urinary incontinence [8]. The pathophysiology behind these complications is that when the ureter has an ectopic orifice in the vagina or urethra, stenosis gradually obstructs urine from free flow, either partially or totally either intermittently or continuously, stasis occurs, and complications follow [8].

Typically, the duplex system is associated with the Weigert-Meyer principle, in which the upper moiety ureter inserts inferomedially and is ectopic while the lower moiety ureter is inserted superolaterally in an orthotopic location [9]. In our case, there was complete duplication of ureters, and the upper moiety ureter was inserted into the vagina, which follows the Weigert Meyer principle of duplex renal systems.

Initial abdomen/urinary tract ultrasound failed to detect the congenital anomaly, as detecting such findings in adults by sonography can be difficult. Sonography is also ineffective for visualizing the entire dilated tortuous ureter and its ectopic orifice [6]. CT urinary track showed the duplex system; however, it did not show the exact ectopic insertion of the ureter. The nephrostogram identified the exact insertion point.

CT urinary tract with intravenous contrast, in the delayed phase, provides superb anatomic detail and diagnostic specificity. Besides its other advantages as fast scanning and the ability to reconstruct three-dimensional images of the urinary tract. This is not only helpful for diagnostic purposes but also when surgical intervention is contemplated, as detailed information about the exact course of the ectopic and normal ureter and visualization of the ectopic ureteral orifice is necessary [6].

Ectopic ureters are managed surgically. The management of a duplex renal collecting system with ectopic insertion depends on the viability of renal parenchyma. Cases with viable kidneys and functional upper moiety will benefit from ureteric re-implantation. On the other hand, permanently damaged renal parenchyma can be treated with partial nephrectomy or heminephrectomy [10].

## Conclusions

This is a case of an incidental finding of a duplicated collecting system with ectopic insertion into the vagina. It highlights the role of the nephrostogram in diagnosing such a condition, especially when the CT urogram is inconclusive about the exact ectopic ureter insertion.
